# Interprofessional Collaboration: The Experience of Nursing and Medical Students’ Interprofessional Education

**DOI:** 10.1177/2333393614560566

**Published:** 2015-01-21

**Authors:** Dawn Prentice, Joyce Engel, Karyn Taplay, Karl Stobbe

**Affiliations:** 1 Brock University, St. Catharines, Ontario, Canada; 2 McMaster University, Niagara Regional Campus, St. Catharines, Ontario, Canada

**Keywords:** education, phenomenology, nursing, medicine, interprofessional education

## Abstract

In this hermeneutic phenomenological study, we examined the experience of interprofessional collaboration from the perspective of nursing and medical students. Seventeen medical and nursing students from two different universities participated in the study. We used guiding questions in face-to-face, conversational interviews to explore students’ experience and expectations of interprofessional collaboration within learning situations. Three themes emerged from the data: the great divide, learning means content, and breaking the ice. The findings suggest that the experience of interprofessional collaboration within learning events is influenced by the natural clustering of shared interests among students. Furthermore, the carry-forward of impressions about physician–nurse relationships prior to the educational programs and during clinical placements dominate the formation of new relationships and acquisition of new knowledge about roles, which might have implications for future practice.

Interprofessional collaboration is considered by many in governments and health care organizations and professions to be critical to the provision of safe, effective, and efficient care. The incorporation of interprofessional collaboration into health care settings affects the everyday practice of nurses. Moreover, preparing nursing students to practice in a collaborative environment demands an understanding of how interprofessional collaboration is developed and mirrored in practice.

Although there is limited evidence to support the effectiveness of collaboration in patient care outcomes, it is thought to improve the quality of health care delivery through the best use of knowledge and skill of health care professionals (Engel & Prentice, 2013). It is also thought to be financially advantageous and socially just because the optimal use of practitioners enables wider access to health care and improved communication among professionals. This can reduce errors and mitigate the human and financial cost of errors. Consequently, groups such as the Committee on Quality Health Care in the United States and the Canadian Nurses Association (2011) have recommended that collaboration is essential among health care practitioners.

Interprofessional collaboration is defined as practice and education where individuals from two or more professional backgrounds meet, interact, learn together, and practice with the client at the center of care. Interprofessional collaboration is seen as potentially a powerful strategy for achieving optimal health outcomes (Green, 2013; Lapkin, Levett-Jones, & Gilligan, 2011). D’Amour, Ferrada-Videla, Rodriguez, and Beaulieu (2005), in their review of the literature, suggest that patient outcomes are improved because of health care collaboration. In addition, better coordination of care is thought to result from increased communication and understanding of each professional’s role.

The perceived significance is reflected by the extent to which interprofessional and intersectoral collaboration is imbedded in international work through the World Health Organization, government documents such as the Romanow (2002) Report, primary health care principles, and various professional standards and position statements such as those by the Canadian Medical Association (2007) and the Canadian Nurses Association (2011). Despite the popularity of collaboration and the amount of literature devoted to interprofessional collaboration in health and education (Lapkin et al., 2011), there continues to be more questions than answers about collaboration. In particular, there continues to be questions about its effectiveness in practice and how it might be taught within prelicensure professional programs (Lapkin et al., 2011).

Although interprofessional education (IPE) dates back to the 1960s and has been the topic of international and national advocacy (Thistlewaite, 2012), there is no specific formula for collaboration or for IPE. Furthermore, there is no solid evidence to suggest that IPE has lasting effects on the interactions of health profession students and graduates. A systematic review of IPE studies conducted by Davidson, Smith, Dodd, Smith, and O’Loughlan (2008) that included studies from the United States, the United Kingdom, Australia, Sweden, and Canada found that the outcomes of IPE were reported as positive. The review identified that significant changes in attitudes and behaviors were reported by participants and that detailed planning and the enthusiasm of organizers were enabling factors in the success of IPE learning. What the review did not establish is whether particular approaches are more effective than others and whether the changes that were reported by students had a lasting impact on practice.

The concern about the lack of evidence that validates the efficacy of IPE is reiterated by Thistlewaite (2012) who suggests that we need longer term evaluation of IPE and its impact on practice, as well as a greater understanding than we currently possess of what collaboration means in practice, to effectively foster and teach interprofessional collaboration. Presently, there are limited frameworks for teaching IPE, most of which focus on specific strategies such as seminars, workshops, and simulation (Reeves et al., 2011). Thistlewaite and Moran (2010) in a systematic review of IPE identified six themes that could be used to guide outcomes for IPE including team roles and responsibilities, communication, learning and reflection, ethics, and attitudes. The limitations in the current knowledge base regarding interprofessional collaboration and how to best acquire skills and knowledge, places greater demands on understanding more about how we learn, teach, and practice collaboration.

## Context

Over the last 2 years, students from a university nursing program have participated in several IPE initiatives with medical students who are from a nearby university that has a satellite medical school situated on the same campus. The nursing program uses a primary health care framework that embraces intersectoral and other collaboration as part of the curriculum but does not have specified interprofessional outcomes or competency statements. The medical students have interprofessional core competencies and mandatory requirements for attendance at IPE sessions. The nursing students attend the IPE sessions voluntarily or because it relates to a specific course.

Interprofessional learning opportunities have included clinical simulations and workshops on a variety of topics (e.g., renal, dermatology, trauma, labor and delivery) and have included many learning approaches including scenarios, simulations, panel presentations, interactions with patients in structured settings, and didactic teaching. No specific learning theory or explicit teaching framework was used for the development of the IPE sessions.

Given the emphasis on interprofessional collaboration, the need to understand more about how interprofessional collaboration is learned, and the future required competencies of graduating nurses, the purpose of this study was to explore the experience of interprofessional learning from the perspectives of nursing and medical students who had been involved in interprofessional collaborative learning.

## Method

Hermeneutic phenomenology was a deliberative choice of method because, as Van Manen (2001) suggests, it gives voice to day-to-day experiences, such as learning in collaboration with others, that is fully and immediately present for participants, as lived. The goal of this method is to articulate the nature of a phenomenon, such as interprofessional learning, not as it is rooted in facticity, but rather as it occurs in the spontaneous life-world and what makes it what it is (Van Manen, 2001) before theory or theorizing. The methods incorporated in Van Manen’s phenomenology are rooted in the Dutch or Utrecht school of phenomenology (Cohen & Omery, 1994) and combine features of both descriptive and hermeneutical phenomenology. Phenomenology demands of the researcher an openness to what emerges from the life-world without the overlay of theory and excessive self-narrative and from the participant an ability to verbalize their experience (Jardine, 1994; Van Manen, 2001) to arrive at a plausible interpretation (Wolcott, 1990).

Using purposive and snowball sampling, medical and nursing students who had attended IPE events within the previous 2 years were recruited by a research assistant (RA) through lists of students who had attended IPE events. A total of 17 students, 7 nursing students in Years 3 and 4 of their program and 10 medical students in Years 1 and 2 of their 3-year program were included in the study.

Once students agreed to participate in the study, the RA engaged them in a conversational interview that was recorded with the permission of the students. The interviews were digitally recorded and transcribed with all identifying information removed, so as to maintain the anonymity of the participants to the researchers. No more participants were recruited after the 17th conversation when it was determined by the researchers that no new themes were emerging. The conversational interviews were largely unstructured, with guiding questions meant to open the dialogue rather than to control it. Some of the guiding questions included the following:

- Please describe your understanding of interprofessional collaboration.
- What are your expectations of interprofessional learning?
- What was it like to experience interprofessional learning? Were there any challenges?
- How do these/will these IPE experiences inform your practice?
- As a result of IPE, what will you do differently?

### Thematic Analysis and Interpretation

Data analysis and interpretation followed the thematic approach outlined by Van Manen (2001). The transcripts were initially read as a whole by three members of the team. Initial impressions were shared among the research team and statements that captured the meaning of the experience were discussed. As a team, the transcripts were then read line by line to identify phrases and sentence clusters that were especially revealing about the phenomenon, which led to further refinement of themes and provided, with notes and observations, a framework for writing the hermeneutic text. QSR International’s (2010) NVivo 9 qualitative software was used to store and organize notes and observations. Opportunities to share emerging findings at professional conferences and with the fourth researcher offered, as Van Manen (2001) suggests, openings for collaborative analyses through which others shared whether the themes and descriptions resonated with their experience and strengthened the interpretation.

### Methods to Ensure Rigor

Phenomenology asks that the researcher come to conversations with the participants fully connected with the experience and engaged in co-creating the understanding of the experience with participants. A phenomenological study that involved students and faculty involves interesting considerations of how conversations might be gathered and the implications that this has for the co-constitution of the experience. The need to balance potential coercion of students into the study because of power imbalances between faculty and students against the involvement of faculty in research conversations presents tension between the method and ethics of conducting a study in which participation and co-creation of experience is voluntary.

To preserve voluntariness and to enrich the sharing of the experience a RA who had a particular interest and experience in interprofessional collaboration was engaged for the research conversations. The RA through biweekly reflexive dialogue with the primary researchers was encouraged to articulate her assumptions and biases throughout the gathering of conversations and how these influenced her conversations with the participants. Through this reflexive dialogue with the RA, the researchers’ journalled these thoughts and impressions that were incorporated into the researchers’ own reflective dialogue during analysis of the transcripts.

### Ethics Clearance

Ethics clearance was obtained from the Research Ethics Board at both universities. The distance between the researchers and the participants was maintained through the engagement of a RA to ensure that participants could freely decide to engage in the study and what to share during research conversations. Informed consent was also obtained by the RA who was the only member of the research team who knowingly had contact with the students during the study. To ensure anonymity of all participants, identifying information was removed and a number was assigned to each transcript. During the write up of the study findings, the participants were described in general terms and numbers were assigned to each quotation so that anonymity of the individual participant is protected. A transcriptionist who signed a confidentiality agreement transcribed the conversations verbatim to honor the words and thoughts of the students.

## Results

Three themes emerged from the data analyses: (a) the great divide, (b) learning means content, and (c) breaking the ice.

### The Great Divide

I was telling to a nursing student we’re working and making a new IP event and he said that on Kidney Day at least, he felt like there was such a massive divide between the med students and nursing students . . . (17)

Within the context of exploring collaborative learning, it seems unusual to describe this experience by using a word such as divide, which appeared within the conversations with students. The Great Divide is an Americanism that commonly refers to the continental divide of North America, a geographic watershed that divides two major drainage systems, each of which is significant but has very different directions in flow and service. When conceptualized in this way, important questions about the experience of collaborative interprofessional learning are raised and how students who aspire to be health professionals can overcome the challenges of different directions to come together and metaphorically move toward a common goal or destination. The divides that the students identified in their experiences were those that arose from the natural clustering of interests, perceptions of roles, perceptions of power, and differences between what was learned in formal situations and clinical settings.

#### At the dance: Power and identity divide

For several of the study participants, the divide that they experienced begins early in the process of learning when students sort themselves according to who they know and shared interests. This clustering establishes, even in beginning interprofessional interactions, that there are dissimilarities among students from different disciplines. For one student, the division was similar to that of elementary school, where the uncertainty of emerging identities drives participants back to where they are comfortable and where they are readily understood by others more like them:

I think it’s a comfort zone thing and I think you’d see it at grade 6 dances where all the girls are on one side and all of the guys are on the other. And we just all kind of sat at tables and I guess naturally like there’s that divide because if you go with people you know you sit with them. (11)

Like, it might have been easier with nursing students all coming from the same class, because you have your allies and you can work together. (4)

Barr (2013) suggests that the retreat to comfort might reflect social identity theories where who we are comes from the membership in social groups; for many of the participants in the group, interprofessional learning seemed to reinforce the need to affirm their membership in a group that is familiar both socially and professionally. One participant described this affirmation as including the perpetuation of stereotypes that have developed, even at this point in the experience of the students:

I would definitely say that there’s a divide in terms of “I don’t know you,” so there’s that, and on a couple of days there’s definitely been “well you’re this” or “you’re that” and “so, this is what your stereotype is and this is what your stereotype is.” (10)

I’ve found the nursing students aren’t interested, not engaged, not interested in participating, not interested in collaborating, they don’t ask questions, maybe they just felt like we were dominating or not welcome to contribute or I don’t know. (15)

For a doctor, I do believe they know more but maybe we know about something that is seen less important, communication right because you know; our physical part of health affects you . . . (7)

Although hermeneutic phenomenology does not seek to compare participants or their respective experiences, the conversations with participants indicated that sometimes the orientation of students toward their own profession and interprofessional learning was so strong that it was immediately evident in the transcripts of the conversations from which the identity and program of the students had been removed. This orientation affected learning and what the students perceived as being learned:

And sometimes I have a questions and I think “oh no, it’s offensive or it’s stupid” but when I started hearing people asking different kinds of questions and the person is like “oh, it’s a good question.” So it was a little challenging to start asking questions that I thought would be inappropriate. (14)

I would just like to reaffirm that I do think that I think a lot us felt inferior because a lot of the medical students would ask questions that to us seemed condescending but really it was maybe just a lack of awareness of our knowledge base and what we are really capable of doing. They would ask us questions like “So we begin with an assessment do you guys know what a head to toe assessment is?” So to us it seemed like those questions were belittling us and that they were very condescending. (6)

For some of the participants, the orientation toward their own profession was already evident in the stereotypes that they had of one another, which seemed to arise out of perceived differences in education and experience and in the value that the students attributed to the learning experience and to respective roles. Despite the rationale offered by the student in the quote above, that the condescending comments from the physician students might be attributed to a lack of awareness rather than purposeful demeaning toward the nursing profession, the general sense from many of the nursing students was the assumption that medical students were smarter, perhaps more academically prepared, and better educated; even for beginning practitioners, this accorded status:

Those assumptions are more or less formed by what you experienced in clinical, and what you know about med programs verses nursing programs—you know they’re a lot harder to get into . . . And in recognizing that in each other and both going “Oh, your program is kind of hard, Like, you know?” (1)

And of course I do believe they know way more, you know they study bio, like microbiology and all of that and it fascinates me all of that stuff more than just communication with patients. (14)

For some of the nursing students, the idea that medical students were smarter was logical because of the decisions that physicians need to make:

. . . there’s that balance and power perception and you know because, they’re ultimately the end decision makers when it comes to making orders and that, right? (1)

But ultimately, physicians are the ones who are responsible. This sole responsibility creates a whole power differential throughout everything. And I feel as though that is a core problem between the two professions. I think part of the way to get around that is to address it front on, because I feel as though a lot of the time it’s sort of swept under the rug in saying “ we are all a team” “we are in this together” but there is still the underlying current of the physician making the final decision. There is definitely a power differential and perhaps acknowledging it was can help relieve some of the division. (7)

Others found this perception difficult and intimidating:

Like, they don’t come up because I don’t particularly like you as a nurse, but somehow I’ve been introduced to the idea that your role is less than mine. (3)

For the participants in this study, power was perceived as social, personal, and intellectual and was related to differences in education, decision making, or the possession of specialized knowledge. This power was expressed in the worth that the participants attributed to the interprofessional events. For the nursing students, the power differential between themselves and the medical students was evidenced by expressions of gratitude at being included in events that involved medical students, “All the lectures were wonderful I mean we did do some learning about problems with the skin, but not to that extent” (5).

The participants who were nursing students experienced the lack of power that is perpetuated, as one participant stated, in “that traditional hierarchy” (9), the participants who were medical students expressed perceptions that were linked to feelings of possessing power, which seemed to arise out of perceptions that they were intellectually or academically superior to the nursing students, or both.

Because I knew the questions to ask I was asking the nursing students but they weren’t necessarily able to answer them. (7)

I feel like they have such concrete answers “oh, I would talk to the physician or administration.” Those just seem to be copycat answers. (17)

For the students in this study, a divide was created, and perhaps even perpetuated, by interprofessional learning because the students sought to define what makes what they do or know different from what students in other professions know or do. This process involves elements of competition and distancing, a process that Green (2013) calls “relative distancing”:

I wonder, if terms of experience, if it [professional competency sessions] could be on the same level, because you come into nursing as an undergraduate degree, whereas in medicine, you *have* an undergraduate degree, so that might not be equal. (9)

It is clear that the opportunity to learn with students from other professional programs is as much as about widening the divide between the groups, as it is about narrowing it. It is about creating different directions for the watershed of knowledge and skills, as much as it is about bringing the different streams together toward a common goal. For the nursing students in the study, there was urgency to narrow the distance, because they perceived that they were not valued, even prior to practice as graduates, and perhaps, to see, in this instance, those who were becoming physicians as somewhat fallible, or at least less as professionals and more as persons, like them:

Like, med students and doctors are a little intimidating, so it was nice seeing them act like human beings and not always having the right answer. So like, I think that could go a long way helping doctors and nurses interact in a professional setting. So like, knowing they are like human beings. It would be definitely helpful understanding each other’s roles. (4)

#### Knowing what and knowing how: Another divide

One of the aims of IPE is to create understanding of one another’s role on an interprofessional team such that a climate of respect is created in which each member of a collaborative team is valued and is able to bring his or her unique knowledge and expertise to patient care (Lapkin et al., 2011). The experience of IPE creates this understanding of roles and contributes to increased capacity for collaboration as students enter their practice areas. The experience of IPE for participants in this study suggested that this might occur, at least minimally, through the interactions of students and presentations that talk about the various roles of professionals in patient care.

. . . because they don’t know, may not know what is expected of us, so now we know what is expected of them, so there’s greater understanding, even empathy wise. (1)

. . . what they can do and what you can do so I think when you educate together, you have more respect for one another’s positions because you you know exactly what they are capable of. (11)

Uhm, because I think some of them are a bit surprised at our knowledge, you know, our prior knowledge about medications and such. So I don’t think they quite understand the kind of knowledge we have, the breadth of knowledge we have . . . (5)

Although some of the participants in the study expressed increased understanding of what the others did in their roles, a troublesome divide existed perhaps between what was learned in the clinical setting about interprofessional collaboration and what was experienced in formalized classroom learning, whether didactic or experientially based:

And I think at in this stage in our training, most interactions are with older experienced nurses, and they obviously treat us differently than somebody who would be our peers in our own level of training our kind of level experience. And then we also observe interactions between physicians and nurses who are different generations from us. So it’s different than the way we might interact when we get to that stage. It’s kind of hard to piece apart what it’s actually going to be like for us, because our interactions are so different. (9)

. . . it is difficult since we are working with preceptors and senior colleagues who have not had this type of training and who do not necessarily have the same value or mentality. That is one challenge trying to operate with our own interprofessional set of values within the greater system. (7)

The experience of the participants suggested that IPE sessions expose students to “the what” of other professions or to the roles of others, “the how” of interprofessional collaboration is learned through observation and interactions within the clinical areas. Learning “the how” in clinical settings from professionals who do not practice interprofessional collaboration potentially sets up a significant conflict or divide between classroom and clinical learning that might serve to reinforce existing assumptions with which students already come to interprofessional work. This, as Thistlewaite (2012) suggests, might lead to questions about how interprofessional learning occurs and the primacy of learning collaboration within the workplace.

### Learning Means Content

For most of the participants, the primary and most significant learning was information that was presented during the IPE event; this was how the worth of the event was determined and how learning was defined:

The presentations were fantastic because we were either talking to patients or looking at equipment . . . it was really helpful because they [the nurses] were basically sharing their experience and giving us the basics of the dialysis machine, what to do. (3)

The main value I have gotten out of them has been the educational content so the kidney information or the dermatological information. That’s been the valuable part for us. (15)

For the participants, the professional identity of the “expert” affected the content and what was learned about the content, suggesting that information and data come imbued with nuances about power and roles:

. . . if a nurse were to present something and a doctor were to present the same thing, it would be entirely different based on the profession. (7)

Historically, medicine is the one that dominates to get away from that stereotype, it almost needs to be a different professional in the situations we are learning about. (16)

Enthusiasm about information gained, particularly that which was new, or opportunities to interact with patients who were willing to share their experiences, generated a somewhat different response from that accorded learning about teams and collaboration:

I like the content because simultaneously we were learning to work with other groups of people, but we’re also getting information, or being taught in things we wouldn’t be taught in other areas. (12)

Although some participants, such as the student in the quotation above, recognized, at least somewhat, that interprofessional learning might mean learning content and solving patient related issues together, it is interesting that others clearly differentiated between what they perceived as content and as process. For these students, interactions with patients, working with unfamiliar technology, or new information was seen as learning, whereas interprofessional collaboration was seen as incidental or secondary to the purpose of the learning events, or possibly, even non learning:

Like, I guess that more of the focus was on learning and so there wasn’t that much emphasis on terms of how you work in a team or how you interact with someone from a different health profession. (9)

Perhaps for these students, interprofessional collaboration might be so taken-for-granted that it is not recognized as needing to be learned or perhaps is sufficiently unimportant that it need not be learned. Both possibilities point to a need for thoughtful intentionality in interprofessional learning and for the need to communicate those intentions clearly to learners, otherwise IPE can assume the status of annoying irrelevance in the face of the competing demands of busy and complex schedules:

. . . it’s [IPE] just another thing that we have to do on top of everything else . . . everyone grumbles “oh I have to go spend eight hours doing something where I should be studying this or reading that.” (13)

### Breaking the Ice

For the participants in the study, collaboration was both a process and an outcome of interaction. The notion of interaction permeated all of the conversations and collaborative interactions were seen largely as a social process that was affected by whether or not the participants felt equal in background to other participants and how learning experiences were structured. For several of the students, the size and composition of groups was important in being able to interact with students who were unfamiliar. Smaller group sizes were helpful in getting to know others and in ameliorating the risks associated with venturing ideas in front of others, particularly those who might judge or who might seem intimidating:

I like more of a one-on-one or a small group; there I am willing to participate more . . . (2)

. . . it’s small enough that . . . everyone’s just mixing . . . (10)

To avoid natural formation of groups along shared interests and experiences, some of the participants expressed the need for the educators to pre-determine and pre-select who should be in groups:

. . . I think it would be better if we could be put in groups where we were forced to interact . . . (13)

For several of the participants, comfort in group interactions came from attempts to establish equitable status with others in the group. The students were keenly aware of differences in their respective clinical and academic exposures, and thus, it seemed important to the students to establish that everyone was a learner and therefore somewhat equal regardless of their respective backgrounds. What was common was that their backgrounds provided a reason to be involved in IPE learning:

. . . we pretty much just have academic knowledge . . . some of the other were all practically based. (12)

. . . you’re both learning and you know when you ask a question that is a little bit silly, like they are learning too so maybe they didn’t know the answer anyways. (11)

Concern with equity was also experienced in relation to how well or how frequently the presenters were representative of all of the disciplines from which the students originate. Equity means inclusion and worth:

Maybe I am biased because I am a nurse, but I think it would have been more effective if there was a nursing perspective to it . . . a lot of it was run by physicians. I did at some point feel marginalized . . . making us feel a little more included . . . like there were different professionals there and not really a lot of nurses . . . (6)

For the students, the social essence of collaboration was expressed in their concern that interprofessional learning needed to acknowledge its relational roots:

I think that, just from my experience and working in with new people from different backgrounds. It takes a while to build up some kind of level of trust? I guess and understanding to feel comfortable, to feel real interaction in a way that’s natural, and because these interactions are quite ad-hoc and very kind of, seems to be a bit impromptu, it’s sort of an unnatural environment to- as much as you act in a way, you know, you’re meeting someone for the first time so you’re open to have people in, you’re more open to saying, “What do you think?” but it’s not to the extent that it would be your colleague that we have with each other because we see each other in a daily basis. (1)

Basic elements of interactions, such as getting to know one another and working together in joint activities were seen as important in building the trust that is important for discussions about roles and to build understanding. Without acknowledgment that collaboration is essentially social, the experience of learning becomes largely parallel interaction and content driven.

## Discussion

The purpose of this study was to understand the experiences of nursing and medical students in interprofessional collaborative learning. Findings suggest that interprofessional collaborative learning is essentially a social experience; as such, its purpose might not necessarily be valued by students, who tend to place a higher value on content, which is equated to learning. The emphasis on content as learning for the medical students might have been related to evaluation methods and progression in their programs, whereas for the nursing students, the experience of content as learning might have been related to the importance that the nursing students attributed to biomedical knowledge in patient care.

The status possessed by biomedical knowledge in patient care was consistent with the perception of power that nursing students attributed to the medical students and that was largely expected by the participants who were medical students in the study. These attributions were especially interesting because they are reflective of traditional hierarchical structures in health care and because the IPE experiences of the students seemed minimally, at best, effective in mitigating stereotypical assumptions about the importance of each other’s roles.

The theme, the great divide, reflects this divisive stereotyping. Furthermore, it suggests that students become polarized even at an early stage in their learning and, that IPE can even serve to solidify these stereotypes and divisions. This is troubling given the purpose of IPE for prelicensure students is for students to understand each other’s roles and what each discipline brings to the patient care team and not to reinforce negative stereotypes. Our finding is not isolated as others have reported that health care students’ views of other students might be based on stereotypes from entry to their program (Hean, Clark, Adams, & Humphris, 2006) as opposed to their experiences (Liaw, Siau, Zhou, & Lau, 2014). One possible explanation for this finding is that the educational events for these students were episodic in nature and were content focused. Offering additional IPE events that focus specifically on interprofessional competencies would provide the students with the opportunity to learn about each other’s roles and examine previously held perceptions.

The medical students were more aware of IPE expectations and saw the IPE learning experiences as falling short, whereas the nursing students, aside from expressed concerns about lack of intermingling between the two groups, did not comment as much on the IPE learning as they did on the content. The medical students in the study had more prior exposure to organized IPE events because this is an inherent component in their curriculum and they, therefore, were more critical than the nursing students and expressed higher expectations of IPE. Conversely, the nursing students had no prior formalized experiences in IPE and, thus, were either unaware of the aims of IPE or its importance or satisfied with its realization in the events. For both groups, content was the overarching benefit in the IPE events.

The finding related to the importance of learning content that is illuminated in this study is consistent with students’ intent to understand their own roles and to focus on learning the content required to be competent, skilled practitioners. More than a periodic cursory exposure to other health care professionals’ roles is necessary to develop an understanding about the roles of the different health care team members and what they contribute to patient care. This continued exposure to the role and contributions of other team members might need to be accompanied by an intentional focus on relational aspects of teamwork for collaboration to occur.

In this study, we have seen that even if students from different disciplines are in the same room with organized group activities to encourage discussion around patient issues, the students found these interactions to be forced and artificial rather than meaningful and engaging, suggesting that a relationship must precede collaboration. As the theme, breaking the ice, suggests, the relational aspect of IPE needs to acknowledged, which implies that communication and trust are foundational to the relationship and that trust is built around valuing differences rather than judging inequities in background skills and knowledge.

Important questions emerge from the study, particularly from the theme, the great divide, which conceptualized a chasm between the two groups of learners. This theme points to factors that discourage interprofessional collaboration such as the natural clustering that occurs within cohorts of students in professional programs who share study times, clinical work, and academic classes within demanding programs and develop a powerful group identification. Within the specific context of interprofessional outcomes, the egocentricity that comes with defining one’s own professional role and its distinct capacity in decisions about patient care potentially competes with a requirement to understand and value the unique contributions of others. Added to this complexity are the differences that students observe between what they are taught (or “the what” of interprofessional collaboration) and what they see or experience in practice, which might suggest how interprofessional collaboration is enacted in practice. This theme raises questions about the best way of achieving interprofessional collaboration and the role of education in IPE, as well as about the timing of education, given the early stereotypes that are formed. It also raises questions about whether the conflicts between what students learn in the classroom and lab and the modeling of experienced clinicians can be overcome effectively through educational programming. Future research should address these questions. Two of the themes, namely, the great divide and breaking the ice, fit in well with both overcoming and seeing this divide.

Communication is an essential component of interprofessional collaboration for all health care professionals to provide patient care that is truly patient centered. If we can overcome the chasm that exists between disciplines and improve understanding of the health care team members’ roles and value what each member contributes to the team, this might contribute to the success of interprofessional collaboration. The best method of accomplishing this raises additional questions—what is the best way of getting there, and at what point in the curriculum?

## Recommendations for Practice

One strategy that arises out of the study is to provide IPE events earlier in the program and to find earlier opportunities for medical and nursing students to interact and have the opportunity to develop relationships. This could include use of small group, case-based activities centered on clinical content where students can actually demonstrate use of their roles. Using this content-focused, small group approach might enhance the value students place on IPE learning. In addition, educators could assign student participants to work in small groups to avoid the natural social clustering and to promote integration. As discussed in the findings, presenters from every discipline should be included in the planning and delivery of IPE events to ensure all disciplines are equally represented. IPE groups should be co-facilitated so that students have the opportunity to view how interprofessional collaboration is practiced. In addition, we will be integrating interprofessional competencies in the nursing curricula.

The findings revealed insights regarding preconceived perceptions about the quality and quantity of the education offered in both medical and nursing education. Based on the perception shared and the participants’ responses to these ideas, it would be essential to integrate knowledge not only of the professional roles but of the educational requirements of each role. This could minimize mistaken perceptions, and allow the students to better understand the educational overlap between the programs and appreciate the differences in education that might lead to better collaboration and less feelings of confusion or condescension.

In addition, the finding, “the great divide,” that indicates the alignment of students was primarily toward their own profession was apparent throughout the participant’s responses and had a significant effect on the learning. Taking this consideration into future planning is a must for IPE. The structure and teaching strategies must be planned in advance to circumvent the tendency of students to stay in their comfort zone close to their academic peers. Planning to ensure equal numbers of learners from different disciplines are in attendanceand then dividing the students into small learning groups that include both professions is a way to start to focus the learning on the IPE objectives.

## Limitations

This study examined the experiences of students from one satellite medical school and one nursing program and did not include students from other health care professions. Therefore, the results might not represent the experiences of all students. This study did not consider the differences that might have existed between the students who had attended one event and those who had attended several. In addition, this study did not differentiate between optional or mandatory events. Examining these aspects could lead to results that provide information about how students experience IPE events when attending more than one event and whether the mandatory or optional nature of the event impacts the experience.

The IPE sessions discussed in this study were not specifically based on one learning theory and neither was one method of teaching used throughout the different sessions. Although the purpose of the study was to better understand the students’ experiences of the IPE sessions currently offered, the planning and implementation of future sessions using a consistent learning theory and similar pedagogies could impact the students’ experiences of IPE revealing different results than found in this study.

## Conclusion

Findings from this study suggest that a chasm exists between nursing and medical students even early in their programs. To overcome this divide, more social interaction opportunities to address and learn about each other’s role are warranted. In addition prelicensure students need to see the benefit of IPE learning for interprofessional learning to be fully appreciated and integrated in to practice.
